# The effects of fish meal substitution by clam meal on the growth and health of Florida pompano (*Trachinotus carolinus*)

**DOI:** 10.1038/s41598-022-11675-x

**Published:** 2022-05-11

**Authors:** H.-Michael Habte-Tsion, Marty Riche, Sahar Mejri, David Bradshaw, Paul S. Wills, Joseph J. Myers, Carlie S. Perricone

**Affiliations:** 1 Department of Aquaculture and Stock Enhancements, Harbor Branch Oceanographic Institute, Florida Atlantic University, Fort Pierce, FL 34946 USA; 2 Cooperative Extension and Aquaculture Research Institute, University of Maine, Orono, ME 04469 USA; 3 Sea Watch International, Ltd., Easton, MD 21601 USA

**Keywords:** Biochemistry, Immunology, Molecular biology, Physiology

## Abstract

A 12-week feeding trial was conducted to evaluate the effects of fish meal (FM) substitution by clam meal (CM, at 10%, 20% and 30% of the diet) on the growth, feed utilization, hepatic antioxidant enzymes, plasma parameters, fatty acid and amino acid composition, and gut microbiome of juvenile Florida pompano, *Trachinotus carolinus*. The results indicated that: (1) juveniles fed 10% and 20% CM had a significantly higher final weight than the group fed the control (0% CM); and the control group also showed significantly lower weight gain, feed intake, protein retention value, whole-body crude protein and total amino acids composition, but higher hepatosomatic index and whole-body crude fat; (2) hepatic peroxide content and superoxide dismutase activity were not significantly affected by the substitution of CM, but it did affect glutathione peroxidase activity, with higher levels found in fish fed 30% CM compared to 0% and 10% CM; (3) plasma total protein, alkaline phosphatase, alanine aminotransferase, and immunoglobulin M showed no significant differences among the treatments; (4) there were no significant differences among treatments in terms of fatty acids composition and microbial diversity. Overall, this study concluded that CM has comparable benefit in the diet of Florida pompano as FM does.

## Introduction

Reducing feed-related costs without affecting the nutritional balance of feed continues to be a priority for the economic sustainability of aquaculture operations and a prime subject for investigation. Historically, fishmeal (FM) and fish oil (FO) from capture fisheries are prime ingredients in aquafeeds. However, due to the decreasing likelihood of increasing wild capture supplies coupled with escalating prices, the continued utilization of these ingredients in aquafeeds looks unfeasible, both practically and economically^1,2^. Therefore, the search and development of new ingredients to replace FM and FO is a high priority for the global aquaculture industry. In view of the actual scarcity of fish-based protein and lipid feedstuffs for use in aquafeeds, processing wastes could be channeled to the aquaculture industry and has the potential for use as seafood by-product ingredients. Although the use of plant-based feedstuffs in aquafeeds has allowed for a reduction in the use of FM and FO, the sector still relies on these prime ingredients in specific formulations for a range of aquatic animals^2^.

The physio-biochemical, immune and antioxidant parameters have been reported as key indicators of fish health in fish nutrition studies^3–8^. Blood parameters are also important indicators of fish health status in response to dietary treatments^3,5–7,9,10^. Liver is one of the target organs in fish nutrition studies with large quantities of unsaturated fatty acids. These lipids are major targets for oxidative damages caused by oxidative stress, which results from an imbalance of reactive oxygen species (ROS)^6,11,12^. Fish are equipped with antioxidant systems to maintain the endogenous ROS at relatively low levels to overcome the damage related to high ROS level^6,11,12^.

Florida pompano (*Trachinotus carolinus*) is one of the promising warm water marine aquaculture species in the United States. Formulated feeds for Florida pompano fundamentally depend on prime feedstuffs such as FM and FO as critical dietary components due to its carnivorous feeding behavior. Therefore, the evaluation of alternative feedstuffs and development of a sustainable feed (both nutritionally balanced and cost-effective) is a key factor for sustainable and economically viable Florida pompano aquaculture in the United States. There are different nutritional studies on this species^13–25^. However, to the best of our knowledge, there is no reported information on the substitution of FM by clam meal (CM) in the diet of this species and its effects on the growth, body composition, hepatic enzymes, gut microbiome, and the health of Florida pompano.

Therefore, this study was conducted to assess the potential use of clam meal (by replacing FM) in the diets of Florida pompano juveniles using growth performance, efficiency parameters, body composition, health parameters (plasma parameters and hepatic antioxidant enzymes) and gut microbiome.

## Materials and methods

### Experimental diets

A control diet, meeting the known requirements for Florida pompano^17^ (with some modification) and three test diets (10%, 20% and 30% CM of the diet, replacing FM from 31 to 3% of the dry diet) were used and are presented in Table 1. Dietary feedstuffs were mixed using a mixer for 30 min followed by the addition of water until adequate consistency for pelleting. The resulting moist dough was screw-pressed through a 3-mm die plate using a laboratory pelletizer. Resulting diet strands were dried in an oven at 60 °C until the moisture content was less than 10%. Thereafter, the diets were broken into appropriately sized pellets, sieved to remove fines, and the pellets were stored at − 20 °C until used during the growth trial. All the diets were run with four replicates (4 treatments × 4 replicate tanks). A subsample of each diet was collected for proximate, amino acid and fatty acid composition analyses (Tables 2 and 3).

**Table 1:** Formulation and analyzed composition of the experimental diets.

Ingredients	Control diet^a^	Test diets^b^ (g/100 g)		
	(g/100 g diet)	10% CM	20% CM	30% CM
Clam meal	–	10.0	20.0	30.0
Menhaden meal	31.2	22.0	12.3	3.0
Soybean meal	20.5	20.5	20.5	20.5
Corn gluten meal	11.8	11.8	11.8	11.8
Wheat gluten	1.5	1.5	1.5	1.5
Blood meal, spray dried	5.0	5.0	5.0	5.0
Shrimp meal	5.0	5.0	5.0	5.0
Dextrin	2.8	2.8	2.8	2.8
Menhaden oil	6.4	7.0	7.5	7.9
Soybean oil	7.4	7.4	7.4	7.4
Sipernat 50	1.0	1.0	1.0	1.0
Mineral premix	1.5	1.5	1.5	1.5
Vitamin premix	0.5	0.5	0.5	0.5
Lecithin	0.1	0.1	0.1	0.1
Stay C-35	0.1	0.1	0.1	0.1
Carboxymethyl cellulose (CMC)	2.0	2.0	2.0	2.0
α-Cellulose	3.3	1.9	1.2	0.0
Total	100.0	100.0	100.0	100.0
**Diet composition (in W/W%)**^**c**^				
Dry matter (%)	92.2	91.2	92.2	91.2
Crude protein*	50.0	50.2	51.1	52.4
Crude fat	8.7	9.1	8.7	9.1
Crude fiber	4.4	3.1	2.5	1.6
Ash	11.3	10.1	9.0	7.7
Digestible energy (kcal/100 g)	375.1	378.7	375.2	376.0

^a^Riche & Williams. Aquaculture Nutrition, 2011, 17: 368–379, with some modification.

^b^Test diets are formulated to have 10%, 20% and 30% CM (clam meal) of the diet while replacing FM. They are isonitrogenous and iso-lipidic with the control diet.

^c^Analyzed by the Experiment Station Chemical Laboratories (ESCL), University of Missouri, Columbia, MO 65211. Results are expressed on an “as is” basis unless otherwise indicated.

W/W% = grams per 100 g of sample.

Crude protein* = %N × 6.25.

**Table 2:** Amino acid compositions of diets.

Amino acids	Clam meal %			
	0%	10%	20%	30%
**Essential amino acids (W/W%)**				
Threonine	1.86	1.94	1.99	2.07
Valine	2.60	2.63	2.58	2.60
Methionine	1.05	1.06	1.04	1.07
Isoleucine	2.11	2.14	2.17	2.21
Leucine	4.63	4.67	4.76	4.68
Phenylalanine	2.42	2.44	2.52	2.51
Lysine	2.92	2.96	2.89	3.02
Histidine	1.35	1.33	1.24	1.18
Arginine	2.69	2.78	2.90	3.09
Tryptophan	0.36	0.37	0.39	0.45
**Conditional essential amino acid (W/W%)**				
Taurine	0.29	0.28	0.27	0.27
**Non-essential amino acids (W/W%)**				
Hydroxyproline	0.40	0.32	0.23	0.14
Aspartic acid	4.24	4.32	4.45	4.56
Serine	1.86	2.11	2.11	2.20
Glutamic acid	7.69	7.63	7.84	7.71
Proline	3.44	3.34	3.36	3.22
Glycine	2.72	2.65	2.40	2.28
Alanine	3.19	3.18	3.08	2.98
Cysteine	0.62	0.67	0.71	0.78
Tyrosine	1.72	1.74	1.87	1.95
Hydroxylysine	0.08	0.07	0.08	0.08
Total AAs (W/W%)	48.31	48.77	48.95	49.13

Analyzed by the Experiment Station Chemical Laboratories (ESCL), University of Missouri, Columbia, MO 65211. Results are expressed on an "as is" basis unless otherwise indicated.

W/W% = grams per 100 g of sample.

Crude protein* = %N × 6.25.

**Table 3:** Fatty acid compositions of diets.

Fatty acid	Clam meal levels (%)			
	0%	10%	20%	30%
C12:0	2.31 ± 0.26	2.53 ± 0.28	3.14 ± 0.24	3.29 ± 0.71
C14:0	5.04 ± 0.80	5.37 ± 0.27	5.60 ± 0.33	5.32 ± 0.78
C15:0	1.81 ± 0.15	1.94 ± 0.19	2.25 ± 0.14	2.42 ± 0.43
C16:0	14.70 ± 0.16	15.39 ± 1.45	15.24 ± 0.51	15.00 ± 2.36
C17:0	1.56 ± 0.16	1.65 ± 0.14	1.96 ± 0.08	2.07 ± 0.36
C18:0	4.13 ± 0.87	4.14 ± 0.33	5.18 ± 0.19	5.47 ± 0.96
C20:0	2.48 ± 0.24	2.64 ± 0.23	3.23 ± 0.25	3.37 ± 0.64
C22:0	2.00 ± 0.17	2.12 ± 0.22	2.58 ± 0.18	2.69 ± 0.52
C24:0	2.07 ± 0.21	1.46 ± 1.28	0.86 ± 1.50	0 ± 0
Total SFA	36.11 ± 3.02	37.25 ± 4.40	40.05 ± 3.43	39.64 ± 6.76
C16:1	5.78 ± 0.18	5.31 ± 0.59	5.95 ± 0.39	5.84 ± 0.20
C18:1	6.90 ± 1.00	7.45 ± 0.46	7.82 ± 0.75	8.23 ± 1.32
C20:1	0.16 ± 0.14	0.16 ± 0.14	0.07 ± 0.12	0.14 ± 0.12
Total MUFA^a^	12.84 ± 1.33	12.92 ± 1.18	13.84 ± 1.26	15.18 ± 3.32
C18:2	21.68 ± 3.87	20.02 ± 0.31	21.47 ± 0.39	20.47 ± 1.67
C18:3 n-3	4.13 ± 0.48	4.55 ± 0.43	0 ± 0	1.57 ± 2.72
C20:2	2.51 ± 0.27	2.70 ± 0.24	3.25 ± 0.25	2.0 ± 1.74
C20:4 n-6 (ARA)	1.10 ± 0.20	1.31 ± 0.03	1.30 ± 0.07	1.33 ± 0.17
C20:5 n-3 (EPA)	11.13 ± 0.69	11.22 ± 0.79	10.91 ± 0.39	11.13 ± 1.41
C22:6 (DHA)	10.12 ± 0.53	9.64 ± 1.02	9.17 ± 0.53	8.67 ± 0.24
Total PUFA^b^	51.59 ± 6.83	50.52 ± 3.59	46.38 ± 2.11	45.23 ± 8.02

Relative percentages of fatty acids (mean ± SD) in diets (control or 0% CM, 10% CM, 20% CM, and 30% CM) fed to Florida pompano (*T. carolinus*) juveniles for 12 weeks.

^a^Sum of monounsaturated fatty acids (MUFA) includes C20:1 for which the percentages are ≤ 0.2% of total fatty acids.

^b^Sum of polyunsaturated fatty acids (PUFA) includes C18:3 n-6, and C20:3, for which the combined percentages are ≤ 0.5% of total fatty acids.

### Experimental system and fish rearing

The use of experimental fish was under scientific research protocols of Florida Atlantic University and Institutional Animal Care and Use Committee (IACUC-A20-29) and complied with all relevant international animal welfare laws, guidelines, and policies. Twenty feed-trained juvenile fish (approximately 6 g initial weight) were stocked into each of the 16 experimental fiberglass tanks (80 L per tank), with each tank serving as the experimental replicate. Diets were randomly assigned to each tank. Following stocking, experimental diets were fed to juvenile pompano 3 times daily to apparent satiation (09:00, 13:00, and 16:00 h), 7 days a week for 12 weeks. All water quality parameters were maintained within the acceptable ranges for Florida pompano, including dissolved oxygen (104.6–121.8%), temperature (27.7–28.3 °C), pH (average 7.5), salinity (average 28.8ppt), alkalinity (average 154.0 mg/L), total ammonia nitrogen (TAN, average 0.0 mg/L), NO_2_-N (average 0.0 mg/L) and NO_3_–N (average 12.1 mg/L) were within acceptable ranges for Florida pompano^26^. A 12-h photoperiod was provided by artificial lighting controlled by a timer. During the 12-week feeding trial, fish were bulk weighed at the start (initial), week-3, week-6, week-9, and week-12 of the feeding trial.

### Sample collection

At the commencement of the growth trial, samples of diets and 50 juvenile fish (~ 5 g each) were collected and stored at − 80 °C pending whole-body proximate, amino acid, and fatty acid analyses. At the end of the growth trial, fish in each tank were sampled after a 24-h fasting and all samples were collected 24-h postprandial. Fish from each tank were anaesthetized with tricaine methanesulfonate (MS-222) at approximately 75 mg/L, and then bulk weighed and counted to calculate the growth parameters. Six fish, from each experimental tank, were anesthetized before blood collection. Blood samples were collected from the caudal vein using heparinized syringes and transferred to a 2 mL lithium heparin tubes and centrifuged at 3000×*g* at 4 °C for 10 min for plasma extraction. The plasma samples were stored at − 80 °C for plasma biochemistry and immune parameter assays. Following blood sampling, the six fish were euthanized with an overdose of MS-222 at 300 mg/L (in culture water) before dissection for collection of organs (liver and part of the mid and hind gut). The liver of three fish per tank (from the euthanized fish) were removed and placed into a 2 mL vial tube and stored at − 80 °C for subsequent hepatic antioxidant enzymes analyses. The intestines were removed from the same three fish and placed into a 5 mL cryovial tube and stored at − 80 °C for gut microbiome analysis. Another five euthanized fish, from each tank, were also stored at − 80 °C and subsequently analyzed for whole-body proximate, amino acid, and fatty acid compositional analyses. We used 12 samples per treatment (three per tank) for the plasma parameters and hepatic peroxide content, and antioxidant enzyme activities and gut microbiome assays. Fish were also individually weighed and measured to calculate the Fulton condition factor (K factor), hepatosomatic and intestinosomatic indices.

### Calculations

The following performance parameters were used to assess the response of the experimental fish to the various dietary treatments:

- Survival (%) = [final population/initial population] × 100
- Weight gain (%) = [(Final body weight – initial body weight)/(initial body weight)] × 100
- Feed efficiency (FE) = weight gain (g)/dry feed consumed (g)
- Protein efficiency ratio (PER, %) = [weight gain (g, wet weight)/protein intake (g, dry weight)] × 100
- Protein retention value (PRV, %) = protein gain/protein intake = [(final body weight (g) × final body protein (%) – initial body weight (g) × initial body protein (%))/(weight of fed diet (g) × protein content of the diet (%))] × 100
- Fulton condition factor, K factor (%) = [fish weight (g)/(fish length, cm)^3^] × 100
- Hepatosomatic index (HSI, %) = [liver weight (g)/body weight (g)] × 100.
- Intestinosomatic index (ISI, %) = [intestine weight (g)/body weight (g)] × 100.

### Proximate composition and amino acid analysis

Proximate composition of diets and whole body were analyzed using standard procedures^27^. The proximate composition and amino acid concentrations of the diets and whole-body sample analyses were carried out by the Experiment Station Chemical Laboratories (ESCL), University of Missouri, Columbia, Missouri (Tables 2 and 5).

### Fatty acid analysis

Diet samples and frozen juvenile fish samples were freeze dried, ground, and homogenized before lipid extraction. Lipids were extracted using modified methods as described by Folch et al.^28^ and Parrish et al.^29^. The fatty acids (FAs) were methylated using methods as described by Lepage and Roy^30^ to produce fatty acid methyl esters (FAMEs) for analysis with gas chromatography-mass spectrometry (GC–MS).

Samples were then analyzed on a Clarus 680/600 T GC–MS (Perkin-Elmer, Waltham, Mass., USA) using a 30 m Thermo Fisher TR-5 general purpose column with a 250-µm diameter. Samples were injected into the column one by one (using an 82-vial autosampler) at a volume of 1.0 µL and heated to 250 °C where it was held for 10 min. Different FAs detected were compared to a 37 component FAME standard (Supelco 37 FAME Mix, Millipore Sigma, Burlington, Mass., USA) with known concentrations for quantification purposes (Tables 3 and 6).

### Physio-biochemical parameters measurement

The content of hepatic peroxide [MDA], activity of hepatic antioxidant enzymes [superoxide dismutase (SOD) and glutathione peroxidase (GPx)], and level of plasma biochemistry [total protein (TP), alkaline phosphatase (ALP), alanine aminotransferase (ALT), and immunoglobulin M (IgM)] were analyzed spectrophotometrically (microplate reader BioTek™, Synergy™ H1, USA) using commercial kits following manufacturer’s protocols (BioVision, Milpitas, CA, USA) according to Habte-Tsion et al.^5–7^.

### DNA extraction

DNA was extracted from the gut of Florida pompano according to Dulski et al.^31^. Gut samples were removed from − 80 °C, allowed to defrost on ice for 30 min and then at room temperature until thawed. Each gut sample was placed into a sterile petri dish for further dissection. A razor blade disinfected with ethanol was used to split the intestines and scrape the gut lumen into a bead-beating tube. Since the fish were fed 24 h prior to sampling and Florida Pompano evacuate three hours after eating, the scrapings were likely more representative of the resident allochthonous bacteria rather than the transient autochthonous bacteria. Care was taken to remove as much fat from the scrapings as possible. DNA was extracted using a Qiagen PowerSoil Pro Kit and then sent to GeneWiz (South Plainfield, NJ, USA) for 16S metabarcoding of the V3-V4 region using proprietary primers.

### Sequence analysis

Demultiplexed raw sequences were processed through a snakemake protocol^32,33^ that uses fastx and trim galore to trim primers and perform quality control^34,35^. Another snakemake protocol based around QIIME2 was used to join and denoise sequences with Dada2^33,36,37^. Annotation was done with Vsearch using a RESCRIPT built version of the 138.1 SILVA database focusing on the V3-V4 region of 16S^38–40^. Sequences were filtered to remove amplicon sequence variants (ASVs) assigned to mitochondria, chloroplast, eukaryotes, and unassigned identification (see Supplementary Table 1 for summary of per sample sequence changes). ASVs were aligned using MAFFT, masked, and used to make a midpoint rooted phylogenetic tree (FASTREE)^41,42^.

### Statistical analysis

Growth, feed utilization, whole-body composition, hepatic peroxide and antioxidants, and plasma health data were statistically analyzed using SPSS version 27 (SPSS, Chicago, IL, USA), after being validated for normality and homogeneity of variances by Shapiro–Wilk and Levene’s tests, respectively. Thereafter, they were subjected to one-way analysis of variances (ANOVA). If a significant difference was identified, differences among means were compared by Tukey’s HSD test. All results were considered significantly different at the level of *p* < 0.05. Results are expressed as mean ± standard error (SE). The relationship between graded dietary clam meal levels and final body weight were subjected to a second-degree polynomial regression analysis using SPSS program.

Permutational analysis of variance (PERMANOVA with 9999 permutations), including a posteriori pair-wise comparisons, was performed on fatty acid profiles of diet and juvenile fish. Each PERMANOVA was tested with one factor: Diet type (control, 10%, 20%, and 30% CM). Assumptions of multivariate homoscedasticity were verified with a PERMDISP test, and data were transformed (arcsine square root) when necessary. To analyze the similarity in fatty acid profiles among different diets, SIMPER analyses were run using a Bray–Curtis similarity matrix with PRIMER 7 (v. 7.1.12) and PERMANOVA + (v.1.0.2).

The outputs from the sequence analysis were imported into RStudio (version 1.4.1106, R version 4.0.4) with the following libraries loaded into the environment: phyloseq (1.34.0), vegan (2.5-7), ggplot2 (3.3.5), plyr (1.8.6), tidyverse (1.3.1), FSA (0.9.1), dplyr (1.0.7), reshape (0.8.8), and DESeq2 (1.30.1)^43–47^. Sequences were filtered to remove low abundance ASVs (< 11 sequences). Alpha diversity, or the diversity within a sample, was calculated with untransformed, filtered ASV counts. It is represented by Shannon diversity (Shapiro–Wilk test p value = 0.2702) because it was statistically correlated with observed ASVs (Spearman Correlation Test p value = 7.016E−16), Fisher diversity (< 3.3E−16) and Simpson diversity (< 3.3E−16). Shannon diversity distribution was normal; analysis of variance (ANOVA) and Tukey tests were used to determine statistical significance between diets. Filtered abundances were square root transformed before beta diversity analysis using Bray–Curtis dissimilarity matrix. This matrix was used to visualize the samples on a principal coordinate of analysis (PCoA) and was imported to PRIMER7/PERMANOVA + to calculate overall and pairwise permutational analysis of variance (PERMANOVA) between diets with 9999 permutations, the unrestricted method, type Type III Sum of Squares, and Monte Carlo adjusted p-values^48^. DESeq2 was used to run indicator analysis at the genus level between the 0% and 30% diets^49,50^. ANOVA, Tukey, and Spearman microbial statistical tests were corrected for multiple testing using Benjamini–Hochberg and all p values less than 0.05 were considered statistically significant. The snakemake, conda environment, R scripts, and R session files can be found on GitHub (https://github.com/aqua-omics/clam_meal_study). Sequences were uploaded into the NCBI database (PRNJ Accession number: PRJNA776864).

### Statement for use of experimental animals

The use of experimental fish was under scientific research protocols of Florida Atlantic University and Institutional Animal Care and Use Committee (IACUC-A20-29) and complied with all relevant international animal welfare laws, guidelines, and policies. The reporting in the manuscript follows the recommendations in the ARRIVE guidelines (https://arriveguidelines.org/).

## Results

### Growth performance, feed utilization and body composition

**Figure 1:**
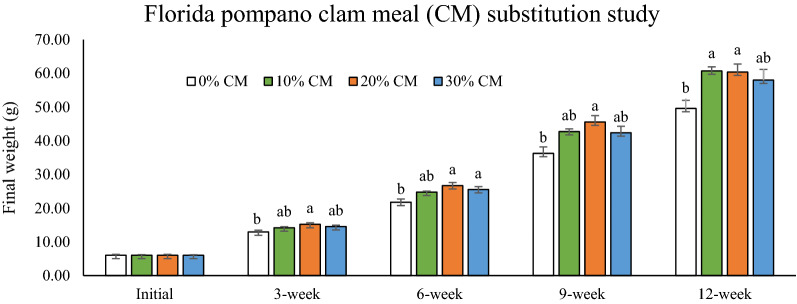
Effects of fish meal (FM) substitution by clam meal (CM, at 10%, 20% and 30% of the diet) on the final weight at different weighing periods of Florida pompano fed the experimental diets for 12 weeks. Values are means with standard errors represented by vertical bars (n = 4). Mean values with different letters are significantly different (P < 0.05).

**Figure 2:**
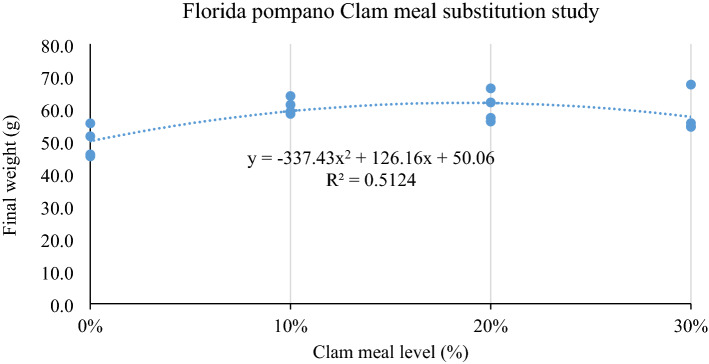
Second-degree polynomial regression analysis of final weight (FW) against dietary clam meal (CM) levels (0%, 10%, 20%, 30% CM of the dry diet) fed to juvenile Florida pompano for 12 weeks.

The survival, growth, and feed utilization (FW, WG, FI, FE, PER, PRV, K factor, HSI, ISI) parameters are presented in Table 4. Survival of Florida pompano was not significantly affected during the growth trial, and it was greater or equal to 97%. Significantly higher (*P* < 0.05) final weight (FW) was obtained in the groups fed 10% and 20% CM compared to the control (FM-based diet fed group). The lowest feed intake (FI) and weight gain (WG) were recorded in fish fed the control diet. Feeding efficiency (FE), Fulton condition factor (K factor) and intestinosomatic index (ISI) were not significantly affected by CM substitution. Fish fed 10% CM showed significantly higher protein efficiency ratio (PER) compared to those fed 30% CM. Protein retention value (PRV) of fish fed 10% CM was higher than the control (*P* < 0.05). The highest hepatosomatic index (HSI) value was found in the control group (*P* < 0.05). During the experimental period, fish were weighed four times (at 3, 6, 9, and 12-week) (Fig. 1). At 3 and 9-week, FW of fish fed 20% CM was higher than the control (*P* < 0.05). At 6-week, FW of fish fed 20% and 30% CM was significantly higher than the control. At 12-week, significantly higher FW was recorded in the groups fed 10% and 20% CM compared to the control. At the end of the 12-week trial, second-degree polynomial regression analysis of FW against dietary CM levels showed that a slight increasing trend of FW up to 20% CM and thereafter back to the level or similar of the control diet (Fig. 2).

**Table 4:** Growth performance, feed utilization and organ indices.

Parameters	Clam meal levels (%)			
	0%	10%	20%	30%
Initial weight (g)	6.03 ± 0.25	6.03 ± 0.20	6.05 ± 0.26	6.03 ± 0.18
Final weight (g)	49.60 ± 2.40^b^	60.70 ± 1.23^a^	60.40 ± 2.35^a^	58.00 ± 3.18^ab^
Weight gain (%)	716.45 ± 20.16^b^	898.65 ± 30.06^a^	876.18 ± 15.06^a^	838.63 ± 34.84^a^
Survival (%)	98.75 ± 1.25	98.75 ± 1.25	97.50 ± 1.44	97.50 ± 1.44
Feed intake (g/fish/day)	1.24 ± 0.02^b^	1.41 ± 0.03^a^	1.48 ± 0.03^a^	1.49 ± 0.05^a^
Feeding efficiency (FE)	0.43 ± 0.03	0.50 ± 0.00	0.45 ± 0.03	0.43 ± 0.03
Protein efficiency ratio (PER, %)	0.70 ± 0.02^ab^	0.77 ± 0.02^a^	0.71 ± 0.03^ab^	0.66 ± 0.01^b^
Protein retention value (PRV, %)	28.55 ± 2.12^b^	36.13 ± 1.71^a^	35.44 ± 1.62^ab^	34.40 ± 1.08^ab^
Fulton condition factor (K factor, %)	1.75 ± 0.03	1.75 ± 0.03	1.74 ± 0.03	1.77 ± 0.04
Hepatosomatic index (HSI, %)	1.74 ± 0.13^a^	1.05 ± 0.10^b^	1.18 ± 0.08^b^	1.24 ± 0.11^b^
Intestinosomatic index (ISI, %)	2.63 ± 0.13	2.35 ± 0.09	2.62 ± 0.09	2.73 ± 0.19

^a,b^Mean values within a row with unlike superscript letters were significantly different (*P* < 0.05).

### Whole-body proximate and amino acid compositions

Whole-body proximate compositions of Florida pompano fed the experimental diets are shown in Table 5. The lowest crude protein and highest crude fat were obtained in fish fed the control diet. Significantly higher ash was found in fish fed 20% CM compared to those fed the control diet.

**Table 5:** Whole-body proximate and amino acid compositions.

Proximate composition (W/W%)	Clam meal levels (%)			
	0%	10%	20%	30%
Crude protein*	34.38 ± 0.92^b^	40.00 ± 1.11^a^	42.24 ± 1.22^a^	43.56 ± 1.40^a^
Crude fat	59.05 ± 0.89^a^	52.60 ± 1.31^b^	49.05 ± 1.44^b^	49.50 ± 0.82^b^
Fiber	0.14 ± 0.02	0.24 ± 0.05	0.29 ± 0.04	3.12 ± 2.63
Ash	7.31 ± 0.36^b^	7.91 ± 0.29^ab^	9.90 ± 0.64^a^	8.43 ± 0.76^ab^
**Essential amino acids (W/W%)**				
Threonine	1.52 ± 0.03^b^	1.66 ± 0.06^ab^	1.81 ± 0.08^a^	1.85 ± 0.05^a^
Valine	1.79 ± 0.04^b^	1.96 ± 0.07^ab^	2.12 ± 0.09^a^	2.17 ± 0.06^a^
Methionine	1.08 ± 0.06	1.10 ± 0.04	1.18 ± 0.05	1.22 ± 0.03
Isoleucine	1.60 ± 0.02^b^	1.75 ± 0.06^ab^	1.88 ± 0.08^a^	1.93 ± 0.05^a^
Leucine	2.55 ± 0.04^b^	2.78 ± 0.11^ab^	3.03 ± 0.13^a^	3.11 ± 0.09^a^
Phenylalanine	1.49 ± 0.03^b^	1.67 ± 0.07^ab^	1.79 ± 0.07^a^	1.85 ± 0.05^a^
Lysine	2.87 ± 0.05^b^	3.11 ± 0.11^ab^	3.43 ± 0.14^a^	3.48 ± 0.10^a^
Histidine	0.80 ± 0.02^b^	0.90 ± 0.03^ab^	0.96 ± 0.04^a^	0.98 ± 0.03^a^
Arginine	2.16 ± 0.08^b^	2.45 ± 0.08^ab^	2.58 ± 0.10^a^	2.66 ± 0.07^a^
Tryptophan	0.24 ± 0.01^b^	0.29 ± 0.01^ab^	0.30 ± 0.02^ab^	0.33 ± 0.02^a^
**Conditional essential amino acid (W/W%)**				
Taurine	0.18 ± 0.03^a^	0.10 ± 0.04^ab^	0.00 ± 0.00^c^	0.02 ± 0.02^bc^
**Non-essential amino acids (W/W%)**				
Hydroxyproline	0.56 ± 0.07	0.63 ± 0.03	0.68 ± 0.05	0.62 ± 0.04
Aspartic	3.29 ± 0.06^b^	3.61 ± 0.13^ab^	3.88 ± 0.16^a^	3.97 ± 0.11^a^
Serine	1.27 ± 0.03^b^	1.40 ± 0.04^ab^	1.54 ± 0.07^a^	1.54 ± 0.03^a^
Glutamic	4.54 ± 0.09^b^	5.01 ± 0.15^ab^	5.46 ± 0.21^a^	5.50 ± 0.14^a^
Proline	1.93 ± 0.04^b^	2.11 ± 0.07^ab^	2.34 ± 0.09^a^	2.22 ± 0.05^a^
Glycine	2.62 ± 0.19^b^	2.96 ± 0.07^ab^	3.16 ± 0.14^a^	3.06 ± 0.10^ab^
Alanine	2.22 ± 0.07^b^	2.46 ± 0.07^ab^	2.68 ± 0.10^a^	2.66 ± 0.06^a^
Cysteine	0.40 ± 0.03	0.39 ± 0.01	0.43 ± 0.02	0.45 ± 0.01
Tyrosine	1.03 ± 0.03^b^	1.11 ± 0.05^ab^	1.24 ± 0.05^a^	1.26 ± 0.04^a^
Hydroxylysine	0.10 ± 0.01	0.10 ± 0.00	0.12 ± 0.01	0.10 ± 0.01
Total amino acids (W/W%)	34.29 ± 0.77^b^	37.60 ± 1.11^ab^	40.70 ± 1.53^a^	41.03 ± 0.96^a^

Analyzed by the Experiment Station Chemical Laboratories (ESCL), University of Missouri, Columbia, MO 65211. Results are expressed on an "as is" basis unless otherwise indicated.

W/W% = grams per 100 g of sample.

Crude protein* = %N × 6.25.

^a,b,c^Mean values within a row with unlike superscript letters were significantly different (*P* < 0.05).

Whole-body total amino acids and essential amino acids (threonine, valine, isoleucine, leucine, phenylalanine, lysine, histidine, and arginine) concentrations were significantly higher in fish fed 20% CM and 30% CM in contrast to fish fed the control diet (Table 5). Higher tryptophan concentration was found in fish fed 30% CM compared to those fed the control diet (*P* < 0.05). Methionine (essential amino acid) was not significantly affected by CM substitution. Significantly higher taurine was found in fish fed the control diet. Non-essential amino acids such as aspartic acid, serine, glutamic acid, proline, alanine, and tyrosine concentrations were higher in fish fed 20% CM and 30% CM in comparison to those fed the control diet (*P* < 0.05). Significantly higher glycine content was found in the group fed 20% CM diet in contrast to those fed the control diet.

### Fatty acid profiles of diets and fish

**Figure 3:**
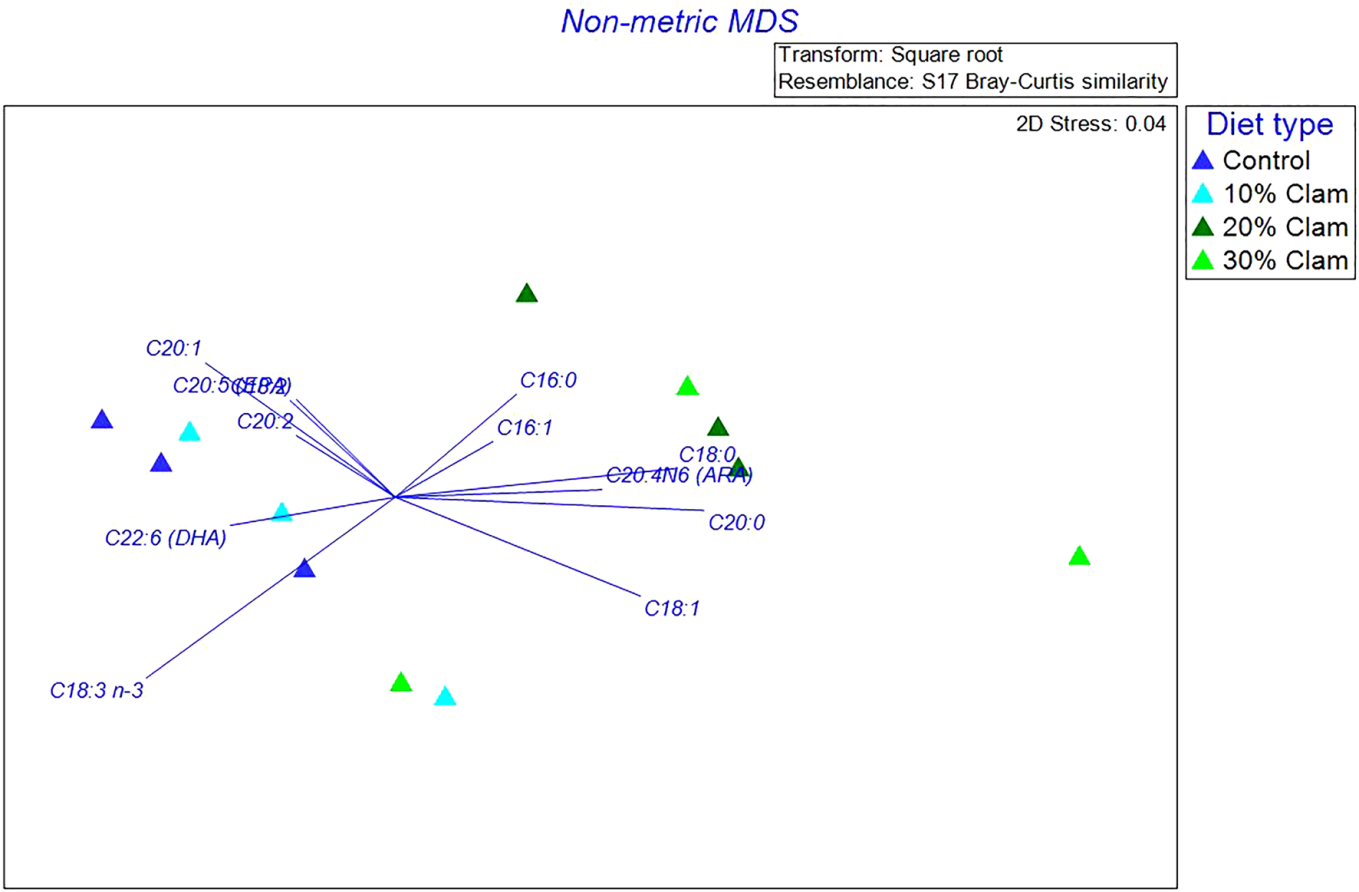
Non-metric multidimensional scaling (n-MDS) of the Bray–Curtis similarity matrix based on the relative abundance of fatty acid profiles associated with diets fed to Florida pompano (*Trachinotus carolinus*) juveniles; control diet (dark blue triangles); 10% clam meal (CM) diet (light blue triangles); 20% CM diet (dark green triangles); and 30% CM diet (light green triangles). The n-MDS shows two main clusters: 1st cluster regroups diet samples from control and 10% CM; and the second cluster regroups diet samples from 20 and 30% CM diets. The arrows length and direction represent the fatty acids responsible for most of the variation.

PERMANOVA analysis on the diets showed that FAs profiles in the four diets are significantly different (Pseudo-F = 3.68, p = 0.028). The FAs profiles from the control diet and the 10% CM diet were similar, they contained higher amounts of polyunsaturated fatty acids (PUFAs), mainly made up of 18: 3 n-3, DHA, and EPA (Table 3). The 20% and 30% CM diets were similar and were characterized by higher percentages of saturated fatty acids (SFA) and monounsaturated fatty acids (MUFA) (Table 3). The n-MDS plot highlights two distinct clusters, with the first one regrouping diet samples from control and 10% CM, and the second one regrouping samples from the 20% and 30% CM (Fig. 3).

**Figure 4:**
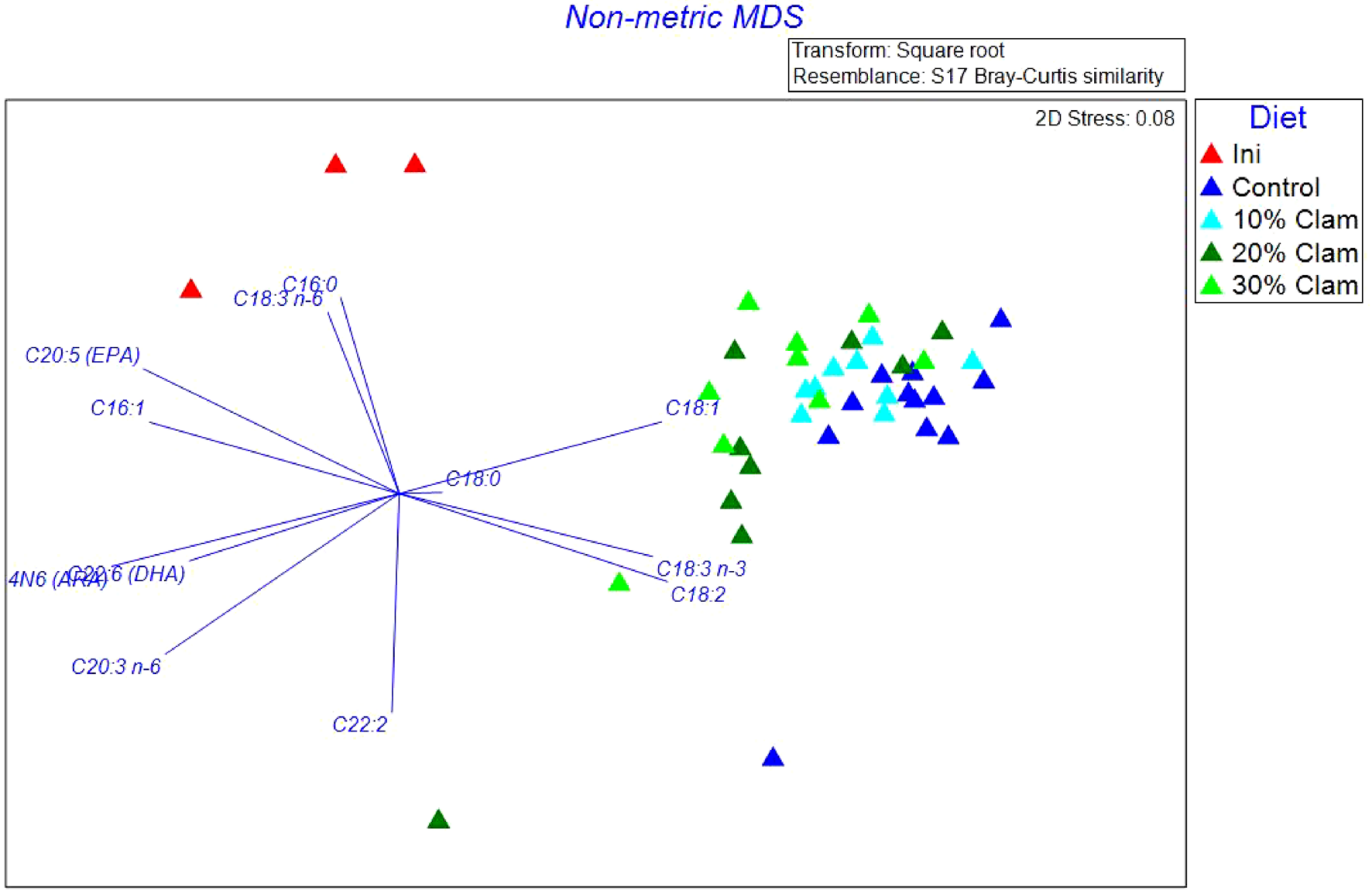
Non-metric multidimensional scaling (n-MDS) of the Bray–Curtis similarity matrix based on the relative abundance of fatty acid profiles associated with Florida pompano (*Trachinotus carolinus*) juveniles at the start of the experiment (red triangles; Initial); juveniles fed a control diet (dark blue triangles); juveniles fed a 10% clam meal (CM) substitution diet (light blue triangles); juveniles fed a 20% CM substitution diet (dark green triangles); and juveniles fed a 30% CM substitution diet (light green triangles). The n-MDS shows two main clusters: 1st cluster regroups all juvenile samples issued from all four diets; and the second cluster (red triangles) regroups initial juvenile samples before the onset of the feed experiment. The arrows length and direction represent the fatty acids responsible for most of the variation.

**Figure 5:**
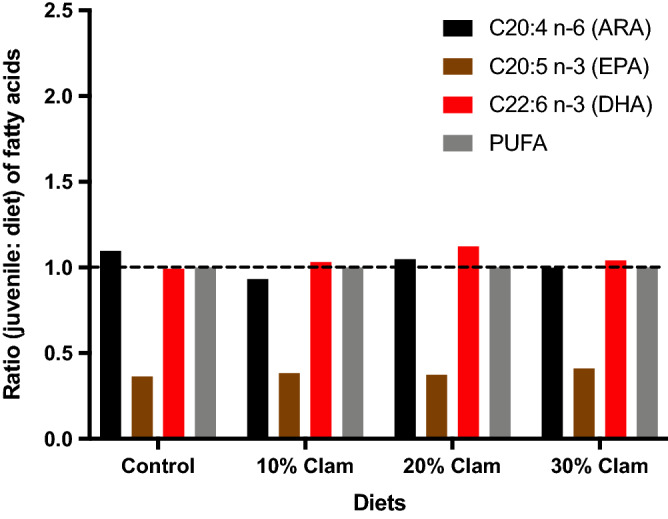
Ratio (juvenile: diet) of fatty acids in Florida pompano (*Trachinotus carolinus*) juveniles after a 12-week feeding trial with a control diet, and a 10%, 20%, and 30% clam meal (CM) substitution into the fish meal control diet. The dashed line (ratio = 1) indicates equal amounts of fatty acids in juveniles and in the diet. ARA, arachidonic acid; EPA, eicosapentaenoic acid; DHA, docosahexaenoic acid; PUFA, polyunsaturated fatty acids.

PERMANOVA analysis showed that FAs in the juveniles were significantly different (Pseudo-F = 9.06, p = 0.001). At the end of the experiment, the FA profiles from juveniles fed all 4 diets were similar, the difference was mainly observed between initial profiles (before administering the diets) and final profiles (at the end of feeding trial) (Table 6). The n-MDS plot shows two distinct clusters based on dietary treatment. The first cluster regroups all samples from the 4 diets, and the second cluster regroups initial samples taken before the feeding trial started (Fig. 4). Essential fatty acids were similar in fish fed all 4 diets, but higher, for DHA, EPA, and ARA in initial fish tissues. The fish to diet [FD] ratio for ARA in the diets containing 10%, 20%, and 30% CM was ≤ 1.0, while the FD ratio for ARA in the FM (0% CM) control diet was > 1.0 (Fig. 5).

**Table 6:** Whole-body fatty acid compositions.

Fatty acid	Juvenile Florida Pompano: Fatty acid content				
	Initial	Control	10% clam meal	20% clam meal	30% clam meal
C12:0	1.32 ± 0.14	1.06 ± 0.23	1.13 ± 0.11	1.36 ± 0.34	1.27 ± 0.15
C14:0	7.74 ± 0.51	4.96 ± 0.39	4.89 ± 0.49	5.30 ± 0.68	5.34 ± 0.65
C15:0	1.41 ± 0.05	1.15 ± 0.14	1.20 ± 0.06	1.36 ± 1.20	1.28 ± 0.12
C16:0	26.71 ± 1.84	23.75 ± 1.30	24.17 ± 0.53	24.28 ± 2.08	24.31 ± 2.17
C17:0	1.20 ± 0.02	1.13 ± 0.14	1.18 ± 0.09	1.32 ± 0.19	1.24 ± 0.15
C18:0	6.62 ± 0.95	6.83 ± 0.95	5.56 ± 0.96	4.78 ± 1.33	5.10 ± 1.09
C22:0	1.46 ± 0.08	1.24 ± 0.23	1.25 ± 0.09	1.44 ± 0.23	1.35 ± 0.12
Total SFA^a^	47.07 ± 4.14	40.93 ± 3.58	40.24 ± 2.42	40.59 ± 5.45	40.69 ± 4.69
C14:1	4.01 ± 0.24	2.62 ± 0.29	2.59 ± 0.21	2.89 ± 0.34	2.89 ± 0.17
C15:1	1.20 ± 0.08	0.98 ± 0.19	1.04 ± 0.08	1.22 ± 0.27	1.15 ± 0.10
C16:1	8.54 ± 0.55	5.58 ± 0.52	5.94 ± 0.45	6.08 ± 0.62	6.11 ± 0.36
C17:1	1.39 ± 0.12	1.01 ± 0.33	1.22 ± 0.12	1.44 ± 0.34	1.35 ± 0.17
C18:1	7.56 ± 1.27	12.05 ± 0.94	11.66 ± 0.98	10.72 ± 1.57	11.49 ± 1.94
C22:1	0.66 ± 0.05	0.72 ± 0.20	0.77 ± 0.27	0.57 ± 0.25	0.82 ± 0.23
Total MUFA	23.36 ± 2.31	22.96 ± 2.48	23.22 ± 2.11	22.94 ± 3.39	23.81 ± 2.97
C18:2	2.33 ± 0.29	8.22 ± 0.58	8.13 ± 1.10	8.04 ± 0.90	7.74 ± 1.17
C18:3 n-6	0.79 ± 0.08	0.52 ± 0.17	0.62 ± 0.05	0.62 ± 0.25	0.71 ± 0.08
C18:3 n-3	2.13 ± 0.14	7.51 ± 0.91	7.97 ± 0.92	7.61 ± 0.76	7.77 ± 0.69
C20:2	1.61 ± 0.33	3.03 ± 0.39	2.82 ± 0.28	2.76 ± 0.32	2.73 ± 0.45
C20:4 n-6 (ARA)	1.75 ± 0.13	1.21 ± 0.13	1.22 ± 0.13	1.36 ± 0.25	1.33 ± 0.13
C20:3 n-6	0.85 ± 0.05	0.65 ± 0.12	0.70 ± 0.05	0.80 ± 0.15	0.76 ± 0.09
C20:5 n-3 (EPA)	7.07 ± 0.64	4.04 ± 0.38	4.29 ± 0.28	4.08 ± 0.42	4.57 ± 0.53
C22:2	0.75 ± 0.02	0.89 ± 0.13	0.84 ± 0.09	0.91 ± 0.08	0.84 ± 0.12
C22:6 n-3 (DHA)	12.27 ± 0.33	10.04 ± 0.55	9.93 ± 0.85	10.30 ± 1.39	9.03 ± 1.02
Total PUFA	29.56 ± 2.02	36.11 ± 3.38	36.53 ± 3.78	36.47 ± 4.53	35.49 ± 4.29

Relative percentages of fatty acids (mean ± SD) in Florida pompano (*T. carolinus*) juveniles at the start of the experiment (referred to in the table as initial) and after 12-week feeding trial when they were fed a control diet, a 10% clam meal diet, a 20% clam meal diet, and a 30% clam meal diet.

^a^Sum of saturated fatty acids (SFA) C13:0 and C20:0 for which the combined percentages are ≤ 0.5% of total fatty acids.

### Hepatic and plasma health parameters

Hepatic peroxide (MDA) and antioxidant enzymes (SOD and GPx) are presented in Table 7. MDA content and SOD activity were not significantly affected by the substitution of FM with CM in Florida pompano fed the experimental diets for 12 weeks. GPx activity of Florida pompano fed 30% CM was significantly higher (*P* < 0.05) than fish fed the control (0% CM) and 10% CM, but not different from those fed the 20% CM diet.

**Table 7:** Hepatic and plasma health parameters.

Parameters	Clam meal levels (%)			
	0%	10%	20%	30%
**Hepatic peroxide and antioxidant enzymes**				
MDA (nmol/mg)	27.29 ± 3.37	21.17 ± 2.56	19.28 ± 1.61	23.04 ± 2.56
SOD (U/mg)	2.37 ± 0.24	1.95 ± 0.42	2.31 ± 0.47	2.08 ± 0.30
GPx (U/ml)	2.29 ± 0.39^b^	2.54 ± 0.35^b^	2.99 ± 0.40^ab^	4.02 ± 0.29^a^
**Plasma health parameters**				
Total protein (g/L)	59.08 ± 1.81	58.47 ± 0.90	56.23 ± 1.77	56.45 ± 1.65
ALP (mU/μL)	13.22 ± 1.30	11.55 ± 1.73	10.96 ± 1.62	9.96 ± 0.77
ALT (mU/mL)	5.07 ± 0.89	4.00 ± 0.89	3.13 ± 0.65	3.57 ± 0.80
IgM (µg/mL)	667.00 ± 16.68	679.20 ± 20.14	653.37 ± 16.39	697.93 ± 23.93

*MDA* Malondialdehyde, *SOD* superoxide dismutase, *GPx* glutathione peroxidase, *ALP* Alkaline phosphatase, *ALT* alanine aminotransferase, *IgM* immunoglobulin M.

^a,b^Mean values within a row with unlike superscript letters were significantly different (*P* < 0.05).

Plasma health parameters including total protein (TP), alkaline phosphatase (ALP), alanine aminotransferase (ALT) and immunoglobulin M (IgM) are presented in Table 7. No significant differences were found among the treatments for any of the plasma health parameters.

### Gut microbiome

**Figure 6:**
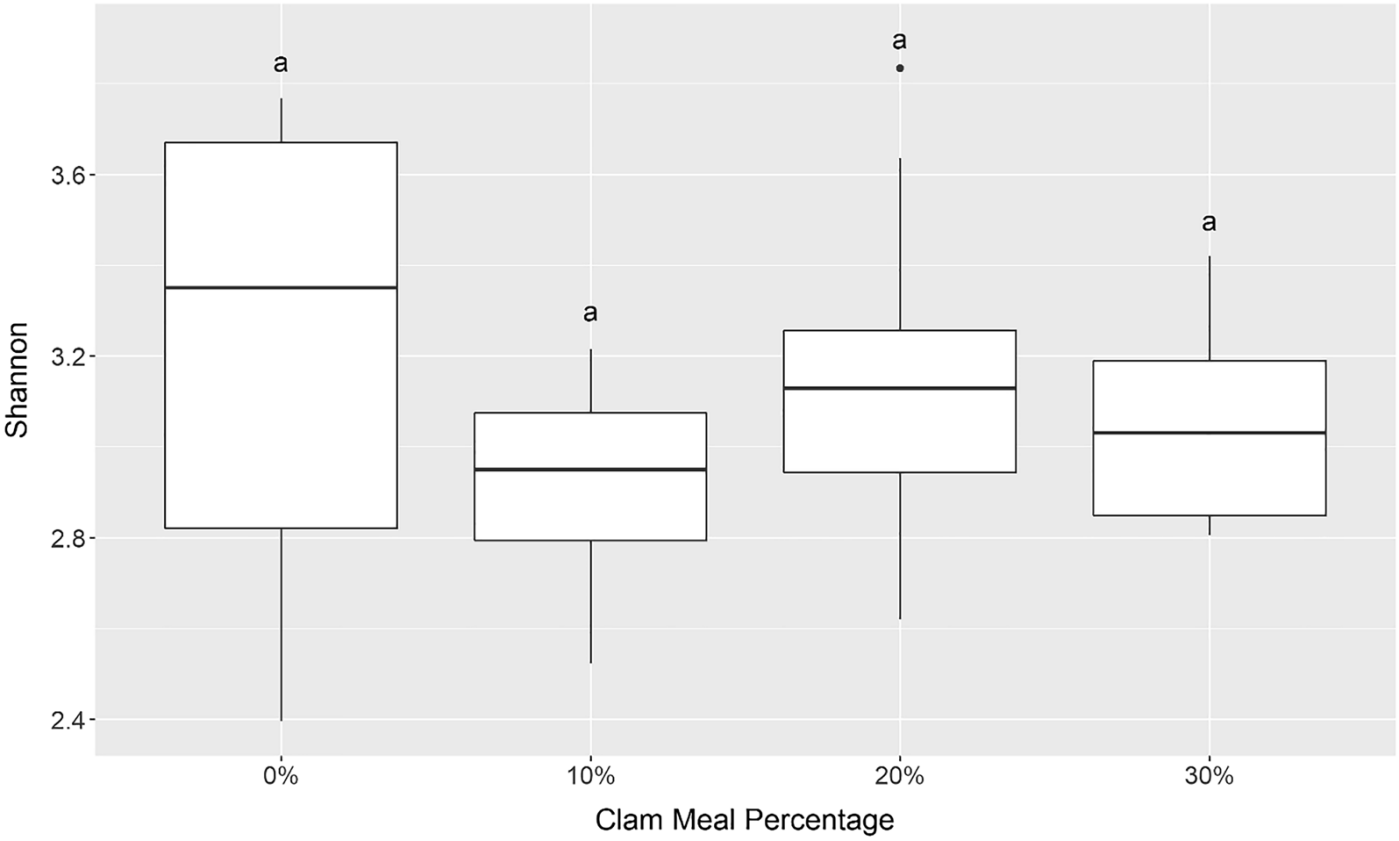
Boxplots summarizing Shannon diversity by diet. Bars denote largest and smallest values within 1.5 times the interquartile range, middle line is the median, ends of boxes are the first and third quartiles. Letters above the boxplots indicate results of pairwise Dunn testing with Benjamini–Hochberg (BH) adjusted p-values. If there is a shared letter between boxplots, that means that they were statistically similar (BH adj. p-value > 0.05).

**Figure 7:**
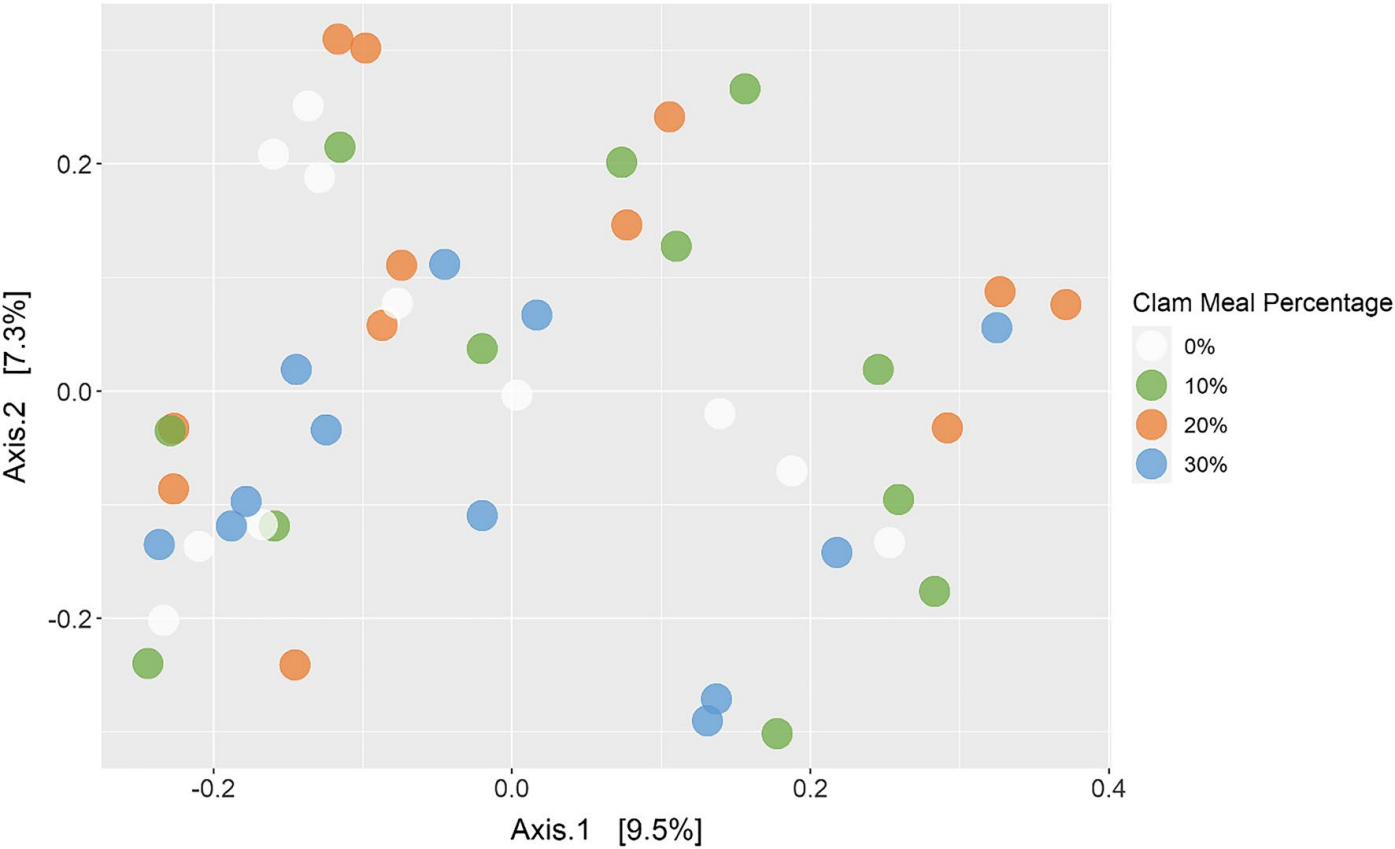
Principle coordinates of analysis of gut samples using a Bray–Curtis dissimilarity matrix after square root transformation. Color represents clam meal incorporation as a percent of the dry diet with white representing 0% (control), green 10%, orange 20%, and blue 30%.

**Figure 8:**
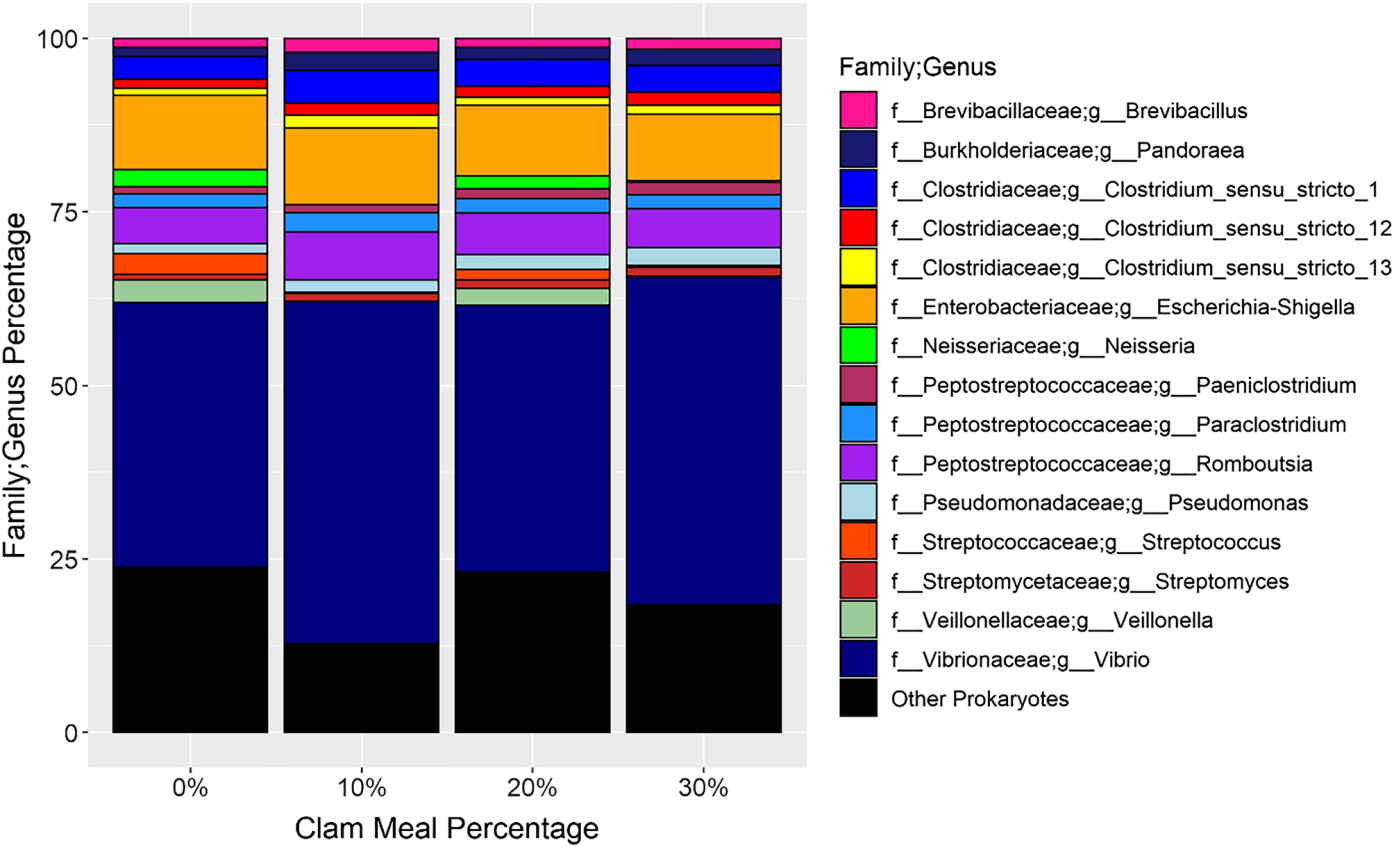
Stacked bar graphs showing the genera that had a mean relative abundance greater than 1% across all samples. Genera and their associated families are represented by color.

After QIIME2 and filtering there were 184,217 sequences distributed across 988 ASVs (see Supplementary Table 2 for summary of taxonomy). ANOVA analysis revealed that Shannon diversity was statistically similar across diets (F-value = 2.0, p-value = 0.13) with an average Shannon diversity of 3.1 ± 0.34 across all samples (Fig. 6). PERMANOVA analysis revealed that there were no statistical differences between diets overall (Pseudo-F = 1.0408, Monte Carlo p value = 0.3756) or pairwise (Supplementary Table 3). This is reflected in the PCoA where there is no discernable pattern in the distribution of sites (Fig. 7). The most common phyla (average > 1%) across all diets were Proteobacteria (65.3 ± 9.79%), Firmicutes (28.1 ± 7.53%), Actinobacteriota (2.59 ± 2.40%), and Bacteroidota (2.00 ± 2.31%) (Supplementary Table 4). The most common genera across all samples were *Vibrio* (43 ± 13%) followed by *Escherichia-Shigella* (10 ± 5.5%) and *Romboutsia* (5.9 ± 2.4%) (Fig. 8). When comparing diets with 0% CM (113 genera) and 30% CM (108 genera), there were 64 common genera between the samples representing 57% of the 0% CM genera and 59% of the 30% CM genera. Of these overlapping genera, the following represented greater than 1% of the 0% CM microbiome and were greater than two times more abundant in the 0% CM samples versus 30% CM samples: *Haemophilus* (0% CM 127 × the 30% CM), *Veillonella* (22x), *Streptococcus* (16x), *Neisseria* (15x), and *Halorhodospira* (2.5x). On the other hand, no genera met those requirements for the 30% CM samples in comparison to the 0% CM samples. DESeq2 Indicator analysis revealed that there were no differentially abundant genera between the 0% and 30% CM diets.

## Discussion

### Growth and efficiency

The results of the present study showed that survival was greater or equal to 97%. Fish fed the 10% and 20% CM diets demonstrated better growth performance such as final weight, PER and PRV in contrast to the FM-based control diet (Table 4). The highest feed intake and weight gain were recorded in the groups fed the CM diets compared to the control. Second-degree polynomial regression analysis of final weight against dietary CM levels also showed an increasing trend 0% up to 20% CM and thereafter back to the level/similar of the control diet (Fig. 2). Studies have been reported on the replacement of FM with different alternative ingredients in Florida pompano diets^14,17–22,24^. However, to the best of our knowledge this is the first study to report the substitution of FM by CM in the diet of Florida pompano. In other fish species, clam by-product supported maximum growth in rainbow trout (*Salmo gairdneri*)^51^ and improved the feed intake in sunshine bass (female White Bass *Morone chrysops* × male Striped Bass *M. saxatilis*)^52^.

### Whole-body proximate and amino acids

FM substitution by CM improved the whole-body crude protein content. The 20% and 30% CM increased the essential amino acids (including threonine, valine, isoleucine, phenylalanine, lysine, histidine, arginine, and tryptophan), non-essential amino acids, and the total amino acids concentrations in Florida pompano (Table 5). The highest crude fat was obtained in fish fed the control diet. These results support the growth performance data of Florida pompano fed the CM diets. Goodrich et al.^51^ conducted an 8-week trial using solid waste recovered from clam processing and evlauated it for its suitability as a replacement protein for FM in rainbow trout (*Salmo gairdneri*) diets and reported (i) clam by-product supported maximum growth and feed conversion and (ii) moisture, protein and lipid contents in carcass were similar among the fish fed the different feeds. Barry et al.^52^ also caried-out two 8-week feeding trials to assess whether the inclusion of sea clam (ocean quahog *Arctica islandica*) processing by-products could improve the intake of reduced fish meal feeds in sunshine bass (female White Bass *Morone chrysops* × male Striped Bass *M. saxatilis*). They reported that the inclusion of sea clam by-products (particularly dried clam fines) improved the intake of a diet with reduced FM, and these by-products appear to be effective feeding stimulants in soy-based diets for juvenile sunshine bass^52^.

### Fatty acids

Fatty acid profiles from juveniles fed CM were similar to juveniles fed the FM diet. One method to determine the juvenile nutritional quality of a feed, at least in terms of essential fatty acids (EFA), is the ratio of specific FA in juvenile tissues to the total FA present in the diet (the fish to diet [FD] ratio). This FD ratio indicates whether a specific FA in the diet is selectively incorporated by the juveniles. To understand which FA might be conserved, deficient, or selectively retained in terms of EFA in Florida pompano juveniles, the FD ratio was calculated for each FA. If the relative proportion of a specific FA in juvenile fish to those in the diet is ≤ 1.0, then the specific requirement for this FA could be considered as met. In contrast, if the relative proportion is > 1.0, then the assumption is that this FA is selectively incorporated by the fish, which may indicate a potential dietary deficiency. The nutritional requirements for ARA appear to have been met in the diets containing 10%, 20%, and 30% CM (FD ≤ 1.0). On the other hand, the FD ratio for ARA in the FM (0% CM) control diet was > 1.0 (Fig. 5). This suggests ARA was selectively retained in juveniles fed the FM-based control diet, indicating a possible ARA deficiency under this dietary regime. This would suggest CM may be a better source of dietary ARA than FM. Few papers have discussed the physiological needs of Florida pompano juveniles in terms of EFA^23,53–55^. In juveniles (43.4 ± 0.2 g) fed six different diets containing different sources of oil (i.e., poultry, soybean oil, MUFA-enriched soybean oil, palm oil etc.), ARA was selectively retained in the brain, but not in the muscle tissues^53^. The authors suggest that ARA is probably not used for energy purposes for juveniles but are most likely required for neural development (reviewed in Mejri et al.^55^).

Requirements for eicosapentaenoic (EPA) seemed to be fully met in all four diets, as indicated by an FD ratio < 1.0 (Fig. 5). That the FD ratio for EPA was ≤ 0.7 suggests that the EPA levels in all the diets fulfilled the requirements for Florida pompano at the juvenile stage, even with CM substitution up to 30% of the dry diet. These results are comparable to a previous study with Florida pompano^53^. Similarly, the docosahexaenoic (DHA) FD ratio was close to 1.0 for all four diets suggesting that all the diets contained satisfactory amounts of DHA. All diets seemed to satisfy the qualitative requirement for DHA (≈ 10% of total FAs) of Florida pompano at the juvenile stage. These values are close to those defined in other studies on Florida pompano juveniles (reviewed in Mejri et al.^55^). In all the diets tested, the overall PUFA FD ratios were equal to 1.0, suggesting that requirements for EFA in general were met when feeding on a FM-based diet and diets incorporating CM over the range 10–30% of the dry diet.

### Hepatic peroxide and antioxidant enzymes

The liver of fish is a tissue with large unstructured fatty acids that present a risk to fish due to oxidative stress^56^. To overcome this risk, fish are equipped with an antioxidant defense system to (i) maintain endogenous ROS at a low level, and (ii) attenuate the oxidative damage resulting from high ROS reactivity^6,7,11,12^. An increase in free radicals causes overproduction of MDA, which is one of the final products of lipid peroxidation in the cells^7^. MDA is well-known as an oxidative stress marker^5–7,57^. In this study, FM replacement by CM didn’t affect the MDA content. In fish, the antioxidant defense system include SOD, GPx, CAT, and GSH^58^, and their enzymatic activities can be correlated with fish nutritional factors^5–8,12,59,60^. In aquatic animals, SOD can catalyze dismutation of superoxide radicals to hydrogen peroxide that can be removed by GPx^12,61,62^. In the present study, SOD activity was not significantly influenced by the substation of CM. Significantly higher activity of GPx was analyzed in fish fed 30% CM compared to those fed the control diet and 10% CM diet, suggesting CM may have improved the antioxidant defense capacity of Florida pompano juveniles. However, further studies are needed to understand the specific mechanisms of the effects of CM substitution on the hepatic antioxidants of fish.

### Plasma health parameters

In fish nutrition studies, blood parameters (including total protein, ALP, ALT, IgM, etc.) are important indicators for health status in response to dietary manipulations^4–7,9,10,60,63,64^. Blood (plasma) total protein is related to the enhancement of digested protein^63–66^. In plasma, the presence of ALP activity is directly related to ALP enzyme release from cells to the extracellular fluids and elevated activity of ALP may occur when there is cell growth, tissue necrosis, or leakage of ALP^7,67,68^. Generally, high plasma ALT activity indicates that a damage/ weakening of normal liver function^63,64,69^. Production of immunoglobulins is a specific immune response following stimulation by an antigen, and the IgM class is the predominant immunoglobulin in fish^4^. In this study, there were no significant differences among the treatments in plasma total protein, ALP, ALT, and IgM levels, suggesting that CM substitution (even at 30% CM) in the diet of Florida pompano has no detrimental health affects as FM.

### Gut microbiomes

Our findings indicate that there was no significant difference between the alpha and beta diversity of gut microbiomes of fish fed a practical-type FM diet versus those fed a diet containing CM as a FM replacement up to 30% of the dry diet (Figs. 6 and 7, Supplementary Table 3). There were also no significant differences in diversity between the different percentages of CM inclusion. Decreases in alpha diversity can be associated with less competition from beneficial flora in response to opportunistic or invading pathogens^70,71^. Thus, it should be viewed as positive that there were no statistical changes between diets. This demonstrates the adaptability of the microbiome to diet changes in Florida pompano. This has also been observed in European sea bass, *Dicentrarchus labrax*^72^ and rainbow trout, *Oncorhynchus mykiss*^71^ given alternative feeds that were combinations of plant and terrestrial animal by-products.

This study is the first study to sequence the gut microbiome of the Florida pompano. Since there were no significant differences between diets, the general microbial ecology of the Florida pompano gut microbiome will be covered here. As seen in other teleost fish studies, Proteobacteria and Firmicutes, Actinobacteria, and Bacteroidetes to a lesser extent, dominated at the phyla level (Supplementary Table 4)^71,72^. *Vibrio* was by far the most dominant genera across all samples (Fig. 8). A meta-analysis of marine gut microbiomes also demonstrated that *Vibrio* was the dominant genus in eleven out of thirty studies, including those focusing on other carnivorous fish such as Atlantic cod (*Gadus morhua*)^73^ and Red drum (*Sciaenops ocellatus*)^74^. Several *Vibrio* species are well-known opportunistic pathogens and can cause Vibriosis which causes mortality through necrosis in internal organs^74^. However, many Vibrio species produce a number of hydrolytic enzymes such as lipase, amylase, cellulase, and chitinase that assist the fish host with breakdown of dietary components^74^. Despite containing opportunistic pathogens, *Escherichia* along with other abundant genera in this study like *Clostridium* and *Streptococcus* can produce short chain fatty acids (SCFA)^75^. SCFA offer beneficial effects on health and growth due to anti-inflammatory, anti-apoptotic, and antimicrobial properties and have been introduced into feeds for this express purpose^75–77^. Another abundant genus was *Romboutsia*, which contains anaerobic, Gram-positive or Gram-variable members that were until recently classified as *Clostridium*^78^. A previous genomic and functional analysis revealed that the type species *Romboutsia ilealis*, isolated from rat gut, could use a wide variety of carbohydrates and express bile salt hydrolase and urease enzymes, but had limited ability to de novo synthesize amino acids and vitamins^79^.

## Conclusion

Our results indicate that Florida pompano juveniles fed 10% and 20% CM had a significantly better final weight, weight gain, protein retention value, and whole-body crude protein, total amino acid composition, and fatty acid composition, compared to fish fed the control diet. Hepatic peroxide (MDA content) and SOD activity were not significantly affected by the substitution of CM. GPx activity was significantly higher in fish fed 30% CM compared to those fed the control and 10% CM diets, suggesting CM may have improved the antioxidant defense capacity of Florida pompano juveniles. Plasma health parameters (total protein, ALP, ALT, and IgM) were not significantly influenced by CM substitution in the diet of Florida pompano. There were no significant differences among treatments in terms of microbial diversity. Overall, this study concluded that CM has comparable benefit in the diet of Florida pompano as FM does.

## Supplementary Information

Supplementary Tables.
