# Supplementary material for: The effects of fish meal substitution by clam meal on the growth and health of Florida pompano (Trachinotus carolinus)
Source: Sci Rep. 2022 May 11;12:7696. doi: 10.1038/s41598-022-11675-x (PMC9095845; doi:10.1038/s41598-022-11675-x)
Supplement: Supplementary file 1 — Supplementary Tables. [file 41598_2022_11675_MOESM1_ESM.docx]

**Supplementary Tables for microbiome work**

**Supplementary Table 1. Per sample sequences breakdown over analysis**

| Sample ID | Raw | Post-Dada2 | Post Filtering Mitochondria | Post Filtering Chloroplast | Post Filtering Eukaryotes | Post Filtering Unassigned | Post Filtering Low Abundance ASVs^1^ |
| --- | --- | --- | --- | --- | --- | --- | --- |
| 01Ga | 36042 | 4388 | 4388 | 4388 | 4388 | 1705 | 1693 |
| 01Gb | 20374 | 12248 | 12248 | 12248 | 12248 | 4921 | 4855 |
| 01Gc | 22692 | 14876 | 14876 | 14876 | 14876 | 6275 | 6197 |
| 02Ga | 23504 | 13515 | 13515 | 13515 | 13515 | 4606 | 4556 |
| 02Gb | 17585 | 11151 | 11151 | 11151 | 11151 | 4868 | 4824 |
| 02Gc | 26295 | 14916 | 14916 | 14916 | 14916 | 6368 | 6301 |
| 03Ga | 19785 | 12681 | 12681 | 12681 | 12681 | 5011 | 4943 |
| 03Gb | 20591 | 11480 | 11480 | 11480 | 11480 | 5680 | 5618 |
| 03Gc | 35773 | 7534 | 7521 | 7521 | 7521 | 1444 | 1428 |
| 04Ga | 21253 | 13420 | 13408 | 13400 | 13400 | 2695 | 2657 |
| 04Gb | 18686 | 11741 | 11726 | 11710 | 11710 | 3241 | 3194 |
| 04Gc | 21218 | 12818 | 12800 | 12800 | 12797 | 2561 | 2504 |
| 05Ga | 15352 | 9425 | 9383 | 9383 | 9379 | 2479 | 2430 |
| 05Gb | 31036 | 21066 | 21059 | 21059 | 21059 | 8559 | 8443 |
| 05Gc | 26248 | 16294 | 16274 | 15973 | 15973 | 3944 | 3847 |
| 06Ga | 15230 | 9731 | 9721 | 9721 | 9721 | 3683 | 3639 |
| 06Gb | 36002 | 4270 | 4270 | 4270 | 4270 | 1015 | 998 |
| 06Gc | 21949 | 13859 | 13859 | 13859 | 13859 | 3520 | 3504 |
| 07Ga | 22412 | 14161 | 14161 | 14161 | 14161 | 4362 | 4296 |
| 07Gb | 24634 | 14881 | 14881 | 14879 | 14879 | 3206 | 3136 |
| 07Gc | 18515 | 11450 | 11450 | 11450 | 11450 | 3308 | 3268 |
| 08Ga | 26603 | 17903 | 17903 | 17903 | 17899 | 7799 | 7721 |
| 08Gb | 20175 | 13393 | 13393 | 13393 | 13393 | 3370 | 3256 |
| 08Gc | 19506 | 12860 | 12860 | 12860 | 12860 | 5166 | 5034 |
| 09Ga | 35565 | 6257 | 6257 | 6257 | 6257 | 2488 | 2403 |
| 09Gb | 19659 | 12026 | 12026 | 12026 | 12026 | 4484 | 4350 |
| 09Gc | 20759 | 12533 | 12533 | 12533 | 12533 | 5653 | 5558 |
| 10Ga | 24172 | 13959 | 13959 | 13959 | 13959 | 5700 | 5577 |
| 10Gb | 16866 | 10315 | 10315 | 10315 | 10315 | 4096 | 3993 |
| 10Gc | 23242 | 14590 | 14590 | 14590 | 14582 | 7093 | 6993 |
| 11Ga | 20864 | 12490 | 12490 | 12490 | 12490 | 4921 | 4799 |
| 11Gb | 16717 | 10066 | 10066 | 10066 | 10066 | 4834 | 4716 |
| 11Gc | 35602 | 3729 | 3729 | 3729 | 3729 | 946 | 912 |
| 12Ga | 18618 | 9241 | 9241 | 9241 | 9241 | 2569 | 2530 |
| 12Gb | 16521 | 9570 | 9570 | 9570 | 9570 | 2839 | 2799 |
| 12Gc | 18463 | 9530 | 9530 | 9530 | 9530 | 2183 | 2161 |
| 13Ga | 14049 | 8225 | 8225 | 8225 | 8225 | 2874 | 2824 |
| 13Gb | 21569 | 13101 | 13101 | 13101 | 13101 | 5961 | 5895 |
| 13Gc | 16164 | 8018 | 8018 | 8018 | 8018 | 1859 | 1808 |
| 14Ga | 13939 | 8146 | 8140 | 8140 | 8140 | 3593 | 3549 |
| 14Gb | 35701 | 7988 | 7988 | 7988 | 7988 | 1438 | 1345 |
| 14Gc | 22210 | 15751 | 15751 | 15751 | 15751 | 2568 | 2525 |
| 15Ga | 23668 | 15715 | 15715 | 15715 | 15715 | 3807 | 3767 |
| 15Gb | 26020 | 18706 | 18706 | 18706 | 18706 | 2823 | 2779 |
| 15Gc | 17487 | 11714 | 11714 | 11714 | 11714 | 2378 | 2361 |
| 16Ga | 25291 | 18276 | 18276 | 18276 | 18276 | 5185 | 5104 |
| 16Gb | 24275 | 17153 | 17153 | 17153 | 17153 | 3533 | 3464 |
| 16Gc | 17742 | 12086 | 12086 | 12086 | 12086 | 3704 | 3663 |
| Total | 1086623 | 579246 | 579103 | 578776 | 578757 | 187315 | 184217 |
| Percent Lost | N/A | 47 | 0.025 | 0.056 | 0.0033 | 68 | 1.7 |
| Percent of Raw | 100 | 53 | 53 | 53 | 53 | 17 | 17 |

^1^ASV stands or amplicon sequence variant

**Supplementary Table 2. Taxonomic breakdown of final abundance table post filtering**

| **Taxonomic Level** | **Total** | **Named** |
| --- | --- | --- |
| Phylum | 17 | 17 |
| Class | 25 | 24 |
| Order | 66 | 65 |
| Family | 116 | 112 |
| Genus | 189 | 160 |
| Species | 236 | 19 |

**Supplementary Table 3. Pairwise permutational analysis of variance results between Diets**

| Clam Meal Percentage Groups | T | Permutational  p value | Unique permutations | Monte Carlo  p value |
| --- | --- | --- | --- | --- |
| 0% CM, 10% CM | 1.0896 | 0.1669 | 9816 | 0.2659 |
| 0% CM, 20% CM | 0.88267 | 0.8939 | 9819 | 0.7144 |
| 0% CM, 30% CM | 1.0163 | 0.4032 | 9798 | 0.4193 |
| 10% CM, 20% CM | 1.0408 | 0.3118 | 9815 | 0.3649 |
| 10% CM, 30% CM | 0.93753 | 0.6696 | 9863 | 0.5743 |
| 20% CM, 30% CM | 1.1339 | 0.0933 | 9823 | 0.2022 |

**Supplementary Table 4. Phylum by Diet**

| Phylum | 0% Clam Meal | 10% Clam Meal | 20% Clam Meal | 30% Clam Meal | Average | Standard Deviation |
| --- | --- | --- | --- | --- | --- | --- |
| Acidobacteriota | 0.176 | 0.178 | 0.276 | 0.301 | 0.233 | 0.236 |
| Actinobacteriota | 3.54 | 1.72 | 3.06 | 2.02 | 2.59 | 2.40 |
| Bacteroidota | 2.36 | 1.40 | 2.61 | 1.63 | 2.00 | 2.31 |
| Campylobacterota | 0.132 | 0 | 0.0978 | 0 | 0.0575 | 0.134 |
| Cloacimonadota | 0 | 0 | 0.114 | 0.260 | 0.0935 | 0.483 |
| Cyanobacteria | 0 | 0 | 0 | 0.312 | 0.0780 | 0.540 |
| Desulfobacterota | 1.86 | 0 | 0.717 | 0.113 | 0.67 | 2.46 |
| Firmicutes | 26.8 | 28.1 | 30.9 | 26.5 | 28.1 | 7.52 |
| Fusobacteriota | 0.874 | 0 | 0.552 | 0.316 | 0.436 | 0.988 |
| FW113 | 0 | 0 | 0 | 0.228 | 0.0571 | 0.396 |
| Gemmatimonadota | 0 | 0.0309 | 0.0287 | 0 | 0.0149 | 0.0614 |
| Halobacterota | 0 | 0.106 | 0 | 0 | 0.0265 | 0.183 |
| Patescibacteria | 0.459 | 0 | 0.288 | 0 | 0.187 | 0.435 |
| Proteobacteria | 63.4 | 68.4 | 61.2 | 68.2 | 65.3 | 9.79 |
| Spirochaetota | 0.315 | 0 | 0 | 0 | 0.0788 | 0.546 |
| Synergistota | 0 | 0 | 0.105 | 0 | 0.0262 | 0.181 |
| Thermotogota | 0 | 0 | 0.0834 | 0.0914 | 0.0437 | 0.212 |
